# *Polygonatum sibiricum* polysaccharides (PSP) improve the palmitic acid (PA)-induced inhibition of survival, inflammation, and glucose uptake in skeletal muscle cells

**DOI:** 10.1080/21655979.2021.2001184

**Published:** 2021-12-07

**Authors:** Jia-Luo Cai, Xiao-Ping Li, Yi-Lin Zhu, Gang-Qiang Yi, Wei Wang, Xin-Yu Chen, Gui-Ming Deng, Lei Yang, Hu-Zhi Cai, Qiao-Zhen Tong, Li Zhou, Mengying Tian, Xin-Hua Xia, Ping-an Liu

**Affiliations:** a Preventive Treatment of Disease Center, The First Hospital of Hunan University of Chinese Medicine, Changsha, Hunan, China; b School of Pharmacy, Hunan University of Chinese Medicine, Changsha, Hunan, China; c Student Affairs Office, Hunan University of Chinese Medicine, Changsha, Hunan, China; d Hunan University of Chinese Medicine, Changsha, Hunan, China; e Tcm and Ethnomedicine Innovation & Development International Laboratory, School of Pharmacy, Hunan University of Chinese Medicine, Changsha, Hunan, China; f The First Hospital of Hunan University of Chinese Medicine, Changsha, Hunan, China; g Department of Scientific Research, The First Hospital of Hunan University of Chinese Medicine, Changsha, Hunan, China; h Preparation Center, the First Hospital of Hunan University of Chinese Medicine, Changsha, Hunan, China; i Yueyang Affiliated Hospital of Hunan University of Chinese Medicine, Yueyang, Hunan, China

**Keywords:** Diabetes mellitus, glucose intolerance, microRNAs, *Polygonatum*, polysaccharides, skeletal muscle

## Abstract

*Polygonatum sibiricum* polysaccharides (PSP) can decrease the levels of fasting blood glucose, total cholesterol, and triglyceride (TG) in hyperlipidemic and diabetic animals. It can also reduce inflammatory cytokines and promote glucose uptake in adipocytes. However, the underlying molecular mechanisms of PSP in improving insulin resistance (IR) in skeletal muscle remain unclear. In this study, palmitic acid (PA) induced an IR model in L6 myotubes. After treatment, cell proliferation was measured using the CCK8. miR-340-3p, glucose transporter 4 (GLUT-4), and interleukin-1 receptor-associated kinase 3 (IRAK3) expression was measured by qRT-PCR. IRAK3 protein levels were measured by Western blotting. Glucose in the cell supernatant, TG concentration in L6 myotubes, and the levels of IL-1β, IL-6, and TNF-α were measured by an ELISA. We found that cell survival, glucose uptake, and GLUT-4 expression in L6 myotubes were significantly suppressed, while lipid accumulation and inflammatory factor levels were enhanced by PA stimulation. Furthermore, PSP treatment markedly alleviated these effects. Interestingly, PSP also significantly reduced the upregulated expression of miR-340-3p in the L6 myotube model of IR. Furthermore, overexpression of miR-340-3p reversed the beneficial effects of PSP in the same IR model. miR-340-3p can bind to the 3′-untranslated regions of *IRAK3*. Additionally, PA treatment inhibited IRAK3 expression, whereas PSP treatment enhanced IRAK3 expression in L6 myotubes. Additionally, miR-340-3p also inhibited IRAK3 expression in L6 myotubes. Taken together, PSP improved inflammation and glucose uptake in PA-treated L6 myotubes by regulating miR-340-3p/IRAK3, suggesting that PSP may be suitable as a novel therapeutic agent for IR.

## Introduction

Type 2 diabetes mellitus (T2DM), one of the most common forms of diabetes, presents a significant global threat to human health [1]. Hyperglycemia is a common symptom of T2DM, and sustained hyperglycemia induces insulin resistance (IR) [2]. IR (i.e., the resistance of target tissues, such as skeletal muscle, fat, and liver, to insulin stimulation) is the pathological basis of T2DM. Skeletal muscle is a major target organ of insulin, and it consumes approximately 80% of postprandial glucose upon insulin stimulation. Glucose metabolic disorders in skeletal muscle cells can affect the metabolism of the whole body. Therefore, improving glucose uptake in skeletal muscle cells plays a significant role in the treatment of T2DM. In recent years, a growing number of studies have found that natural medicines have high safety profiles and multiple pharmacological functions for treating T2DM (e.g., improving blood glucose control, reducing complications, and favorably affecting cardiovascular functions) [3–5]. *Polygonatum sibiricum* is a traditional medicinal herb and a functional food in China [6]. Increasing evidence indicates that *P. sibiricum* or Chinese medicine compounds containing *P. sibiricum* can regulate blood sugar levels in animal models [7,8]. *P. sibiricum* polysaccharides (PSP), important active compounds in PS, are responsible for a broad spectrum of functions, including improved immunity and anti-fatigue, anti-oxidant, anti-aging, and anti-inflammatory effects [9]. Our previous study found that PSP reduces inflammatory cytokines and promotes glucose uptake in adipocytes [10]. However, the mechanism underlying PSP-induced improvement in glucose metabolism in the skeletal muscle remains unknown.

Recently, microRNAs (miRNAs) have attracted global interest in complex human diseases, including T2DM [11]. These small (18–25 nucleotides) endogenous, single-stranded, non-coding RNA molecules act as regulators of mRNA degradation and inhibit the translation of distinctive areas of target proteins by binding to the 3ʹuntranslated regions (3-UTRs) of target mRNAs [12]. Emerging evidence suggests that miRNAs are involved in T2DM pathogenesis. For instance, Zhou et al. [13] found that miR-106b, miR-27a, and miR-30d play crucial roles in regulating glucose metabolism by targeting glucose transporter 4 (GLUT-4) expression in T2DM skeletal muscle cells. GLUT-4 is responsible for the majority of glucose disposal following meals in skeletal muscle. Therefore, whether PSP improves glucose metabolism in skeletal muscle cells via miRNAs remains to be determined.

In this study, we assumed that PSP can improve cell proliferation, inflammation, and glucose transport by regulating the expression of miRNAs. We first investigated the effect of different PSP concentrations on the growth of L6 myotubes. Then, an L6 myotubes IR model was established to investigate the ability of PSP to improve glucose and lipid metabolism. The underlying mechanisms of PSP-induced improvement in glucose and lipid metabolism through miRNAs in L6 myotubes were investigated. This study aimed to explore the potential of PSP to counteract muscle IR, thus providing a theoretical foundation for the clinical application of PSP.

## Materials and methods

### Cell culture and IR model establishment

The rat skeletal muscle cell line L6 myoblasts were purchased from the Cell Bank of the Chinese Academy of Sciences (Shanghai, China). L6 myoblasts were cultured in Dulbecco’s modified Eagle’s medium (DMEM; 5 mM glucose; Gibco) supplemented with 10% (v/v) fetal bovine serum (FBS; Invitrogen) and 100 U/mL penicillin-streptomycin (Sigma) at 37°C in a humidified atmosphere with 5% CO_2_. Once cultured skeletal muscle cells reached 70–80% confluence, differentiation was induced by DMEM (Gibco) supplemented with 2% (v/v) horse serum and 100 U/mL penicillin-streptomycin. The cell culture medium was refreshed every other day and the cells were cultured for six days until myoblasts changed to myotubes. Most myocytes differentiated into multinucleated myotubes and were easily identified as muscle cells, indicating successful differentiation. Successfully differentiated L6 cells with aligned and fused myotubes were used in subsequent experiments. L6 myotubes were cultured in DMEM (25 mM glucose) supplemented with 2% (v/v) FBS and 100 U/mL penicillin-streptomycin at 37°C in a humidified atmosphere with 5% CO_2_. L6 myotubes were treated with 0.5 mM palmitic acid (PA; Sigma) for 24 h to establish the IR model according to a method reported by Wang et al. [14]. Briefly, PA was dissolved in ethanol and diluted to 1:10 in fatty acid-free (>98%) bovine serum albumin (BSA; Sigma; BSA final concentration: 2% w/v). Then, PA-BSA was diluted to 1:10 in 1% FBS-DMEM containing 2% (w/v) BSA.

## PSP source and treatment

PSP (extracted from *P. sibiricum*; China name: Huangjing; purity > 90%) was purchased from Shaanxi Undersun Biomedtech Co., Ltd. (http://www.undersun.com.cn, Shanxi, China). The plant name of *P. sibiricum* was checked at http://www.theplantlist.org. PSP was dissolved in 0.1 mol/L phosphate-buffered saline (PBS) to prepare 1 mg/mL PSP solution. For PSP treatment, 1 mg/mL of PSP solution was diluted to the desired concentration with 0.1 mol/L PBS. The control group received the same volume of 0.1 mol/L PBS. Subsequently, L6 myotubes were divided into several groups: control, PA (model), PA+PSP (50 μg/mL), PA+PSP (100 μg/mL), PA+PSP (250 μg/mL), and PA + metformin groups. In the PA+PSP groups, PA-treated L6 myotubes were treated with different concentrations of PSP for 48 h. In the PA + metformin group, PA-treated L6 myotubes were treated with 0.2 mM metformin (Sigma) for 48 h, which acted as a positive control to compare the treatment effect of PSP. Before Oil Red O staining and glucose and triglyceride (TG) concentration analysis, L6 myotube cells in all groups were stimulated with 100 nM insulin for 30 min.

## Cell survival and apoptosis assay

L6 myotube survival was evaluated using a Cell Counting Kit-8 (CCK8; Beyotime, Shanghai, China) [15]. Briefly, L6 myotubes were digested and seeded into 96-well plates at density of 1 × 10^4^ cells/100 μL/well in triplicate after treatment or transfection, and incubated in a humidified incubator with 5% CO_2_ at 37°C. Next, 10 μL of CCK8 reagent was added to each well after 0, 24, 48, and 72 h, and further incubated for 2 h at 37°C. Absorbance was measured at 450 nm using a microplate reader (Bio-Rad). In addition, the apoptosis of L6 myotubes was measured using the Annexin V-FITC Apoptosis Detection Kit (BD Bioscience, San Jose, CA, USA) using a BD FACSCanto Flow Cytometer (BD Bioscience) [15].

## Oil red O staining

Lipid accumulation in L6 myotubes was detected based on Oil Red O staining [16]. Culture media were removed completely, L6 myotubes were washed with PBS, fixed in 10% (v/v) formalin for 30 min, and then rinsed with PBS. Next, L6 myotubes were stained with freshly prepared Oil Red O working solution for 60 min at 25°C. The staining solution was discarded, and the L6 myotubes were rinsed with PBS. Finally, the L6 myotubes were visualized under a light microscope.

## Glucose and TG concentration analysis

The L6 myotubes were cultured in six-well plates. After appropriate treatment, glucose and TG concentrations in the cell supernatant and L6 myotubes, respectively, were measured using enzyme-linked immunosorbent assay (ELISA) kits from Ziker Biological Technology Co., Ltd., according to the manufacturer’s instructions (cat. nos. ZK-R4616 and ZK-R3310 (Shenzhen, China) [10]. Glucose uptake efficiency was negatively correlated with glucose concentration in the supernatant. Based on the ELISA kit instructions, 50 μL of specimen and 100 μL of HRP-labeled detection antibody were added and incubated at 37°C for 60 min. Then, 50 μL of substrates A and B were added and incubated at 37°C in the dark for 15 min, and 50 μL of the stop solution was added to each well. The optical density (OD) was measured at 450 nm using a microplate reader (Bio-Rad). The concentration of each sample was calculated based on the standard concentrations. Each experiment was repeated three times. Finally, the concentration of each sample was normalized to the total amount of protein in each well.

## Bioinformatic analysis

The Gene Expression Omnibus (GEO, https://www.ncbi.nlm.nih.gov/geo/) dataset search engine was used for the miRNA expression profiling of rat skeletal muscle. The keywords ‘miRNA’ OR ‘microRNA’ OR ‘miR’, ‘skeletal muscle’, ‘diabetes’, and ‘rat’ were used to search for potential studies. GEO profile (GSE68225) was identified [17]. This dataset obtained after final screening contained information about miRNAs derived from the skeletal muscle of diabetic rats, thus matching our requirement. The GSE68225 profile represented six disordered miRNAs in six normal and six IR skeletal muscle rats (SD rats, male, 1 year) by GEO2R analysis. Then the potential target mRNAs of miR-340-3p was analyzed using Targetscan 7.0 [18], miRDB [19], and miRwalk [20]. Additionally, the keywords ‘mRNA’, “’skeletal muscle’, and ‘diabetes’ were used to search for potential studies. The GEO profile (GSE29221) was identified [21]. Abnormally expressed mRNA between male patients with type 2 diabetes and non-diabetic male patients was analyzed using GEO2R.

## Luciferase reporter assay

In brief, the wild-type and mutant interleukin-1 receptor-associated kinase 3 (IRAK3) 3ʹ-UTR sequences were cloned into the luciferase reporter vector psi-CHECK2 (WT-IRAK3 and Mut-IRAK3). Then, WT-IRAK3 plus miR-340-3p mimics or NC mimic and Mut-IRAK3 plus miR-340-3p mimics or NC mimic were transfected into 293 T cells using Lipofectamine 2000 (Invitrogen). Forty-eight hours after transfection, the luciferase assays were performed using the dual luciferase reporter assay system, and the ratio of *Renilla*/firefly luciferase (Promega) was calculated.

## Cell transfection

Rno-miR-340-3p mimics/inhibitors and a negative control mimic and inhibitor (NC mimic and inhibitor) were designed by GenePharma (Shanghai, China). L6 myotube transfections were performed using Lipofectamine 2000 (Invitrogen), according to the manufacturer’s instructions [22]. In brief, a total of 100 nmol/L miR-340-3p mimics/inhibitors or NC mimics/inhibitors were transfected into the L6 myotube at 37°C for 24 h, and then used for subsequent experiments.

## Quantitative real-time PCR (qRT-PCR)

Total RNA was extracted from L6 myotubes using TRIzol reagent (Invitrogen). The stem-loop-specific primer method was used to measure the expression levels of miR-340-3p, and qRT-PCR was performed using TaqMan miRNA assays (Applied Biosystems). All PCRs were performed using an ABI PRISM® 7500 Sequence Detection System (Applied Biosystems) with a SYBR® Premix Ex Taq™ Kit (TaKaRa). PCR primers were purchased from GenePharma with the following sequences: rno-miR-340-3p forward, 5ʹ-ACACTCCAGCTGGGTCCG TCTCAGTTACTTʹ and reverse, 5ʹ-CTCAACTGGTGTCGTGGA-3ʹ; and U6 forward, 5ʹ-CTCGCTTCGGCAGCACA-3ʹ and reverse, 5ʹ-AACGCTTCACGAATT TGCGT-3ʹ. U6 were used as an endogenous control for miR-340-3p expression. The fold-change in expression was calculated using the 2^−ΔΔCT^ method[23].

## Inflammatory cytokines level assay

Inflammatory cytokines, including interleukin-1β (IL-1β), IL-6, and tumor necrosis factor-α (TNF-α) levels in culture supernatants were measured using an ELISA according to the manufacturer’s protocol (Multi Sciences, Hangzhou, China). Finally, the concentration of each sample was normalized to the total amount of protein in each well.

## Western blot

The operation steps of Western blotting were described in our previous research [10]. The primary antibodies used were as follows: anti-IRAKM (1:200, ab8116, Abcam, Cambridge, MA, USA) and anti-GAPDH (1:10,000, EPR16891, Abcam). GAPDH served as a reference protein.

## Statistical analysis

All statistical analyses were performed using the Statistical Package for Social Sciences (SPSS) version 19.0 (IBM Inc.). All data are normally distributed and are provided as means ± standard deviation (SD). Differences were analyzed using one-way analysis of variance (ANOVA), followed by Tukey’s post-hoc test. Statistical significance was set at *p* < 0.05.

## Results

### The effect of PSP on the cell survival rate of L6 myotubes

**Figure 1.**
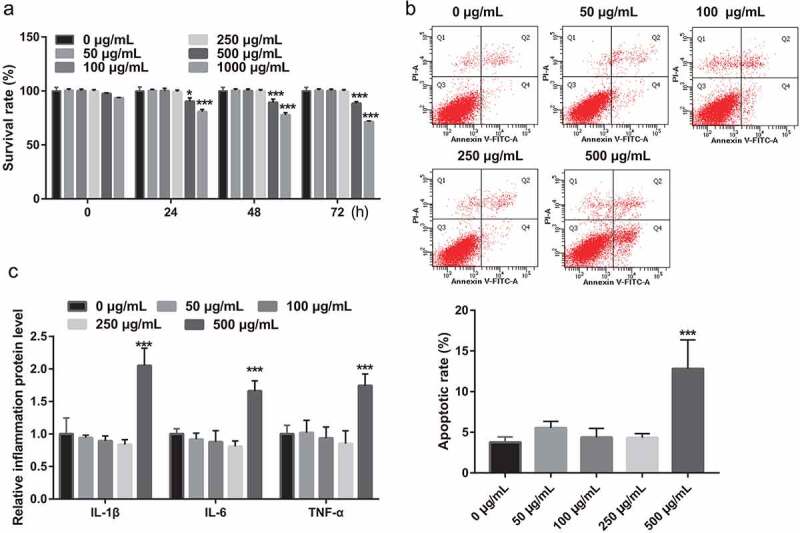
Effect of PSP on the survival, apoptosis, and inflammation of L6 myotubes. (a) The effect of different PSP concentrations on the cell survival of L6 myotubes as assessed by CCK8 assay (*P < 0.05, **P < 0.01, ***P < 0.001 vs. 0 μg/mL). (b) The effect of different PSP concentrations on the apoptosis of L6 myotubes as assessed by Flow Cytometer. (c) The levels of IL-1β, IL-6, and TNF-α in the cell supernatant as measured by ELISA. PSP: *Polygonatum sibiricum* polysaccharide; PA: palmitic acid; CCK8: Cell Counting Kit-8

To study the effect of PSP on the cell survival rate, apoptosis, and inflammation of L6 myotubes, different concentrations of PSP were added to treat L6 myotubes. Compared to the untreated group, PSP treatment did not affect the survival of L6 myotubes at concentrations below 250 μg/mL, but significantly suppressed their survival at concentrations above 500 μg/mL, after treatment at 24, 48, and 72 h (Figure 1a). Furthermore, compared to the untreated group, PSP treatment did not affect the apoptosis and the levels of IL-1β, IL-6, and TNF-α of L6 myotubes at concentrations below 250 μg/mL, but significantly promoted the apoptosis and the levels of IL-1β, IL-6, and TNF-α at concentrations of 500 μg/mL (Figure 1b and c). Therefore, PSP concentrations of 50, 100, and 250 μg/mL (a nontoxic dose range) were selected for subsequent experiments.

### PSP improves glucose uptake, TG metabolism, and inflammation in PA-induced L6 myotubes

**Figure 2.**
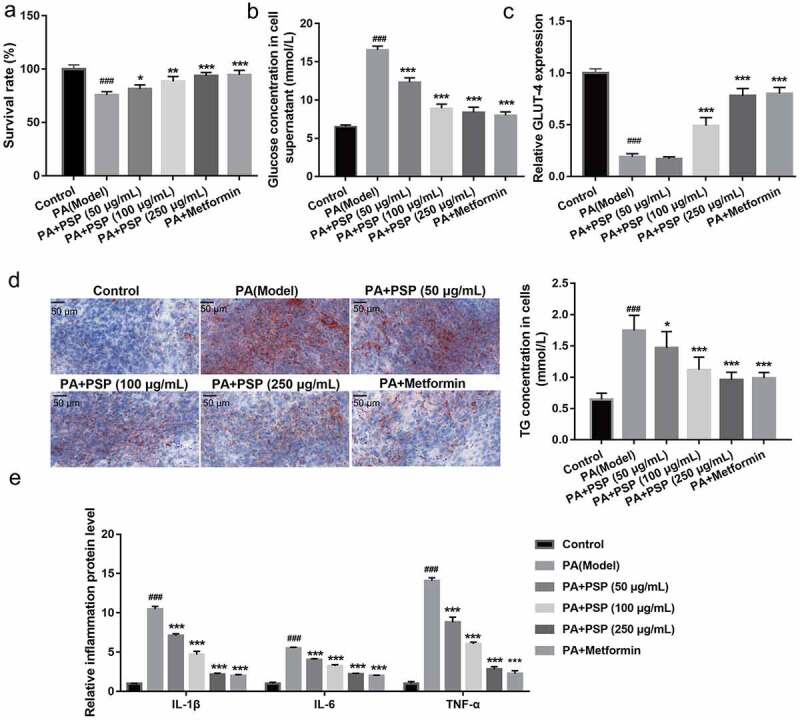
PSP improves the survival, glucose and TG metabolism, and inflammation in the PA-treated L6 myotubes. (a) The effect of PSP on the survival of the PA-treated L6 myotubes. (b) The glucose concentration in the L6 myotubes supernatant were measured by glucose detect kit after stimulated with 100 nM insulin after treatment 48 h. Glucose uptake efficiency was negatively correlated with glucose concentration in the supernatant. (c) The GLUT-4 expression in L6 myotubes as measured by qRT-PCR. (d) The effects of PSP on lipid accumulation as assessed by Oil Red O staining (left, ×400). The TG concentration in the L6 myotubes as measured by TG detect kit after treatment for 48 h (right). (e) The levels of IL-1β, IL-6, and TNF-α in the cell supernatant as measured by ELISA. *^###^P* < 0.001, control *vs* PA model group; **P < 0.01 and ***P < 0.001, treatment group *vs* PA group. PSP: *Polygonatum sibiricum* polysaccharide; PA: palmitic acid; TG: triglyceride

Additionally, to explore the effects of PSP on the survival, glucose uptake, TG metabolism, and inflammation of PA-induced L6 myotubes, PA-stimulated L6 myotubes were treated with different concentrations of PSP. PA stimulation significantly inhibited the survival of L6 myotubes compared with that of the control group, while treatment with 50, 100, and 250 μg/mL PSP and metformin improved cell survival in the PA-treated L6 myotubes (Figure 2a). The effects of PSP on glucose and TG metabolism in PA-induced L6 myotubes were further investigated. Compared to the control group, the levels of glucose, IL-1β, IL-6, and TNF-α in the cell supernatant (Figure 2b and e) and TG concentration in L6 myotubes (Figure 2d) were significantly increased, whereas GLUT-4 mRNA expression was significantly decreased in the PA-induced group (Figure 2c).

### PSP inhibits miR-340-3p expression in PA-induced L6 myotubes

**Figure 3.**
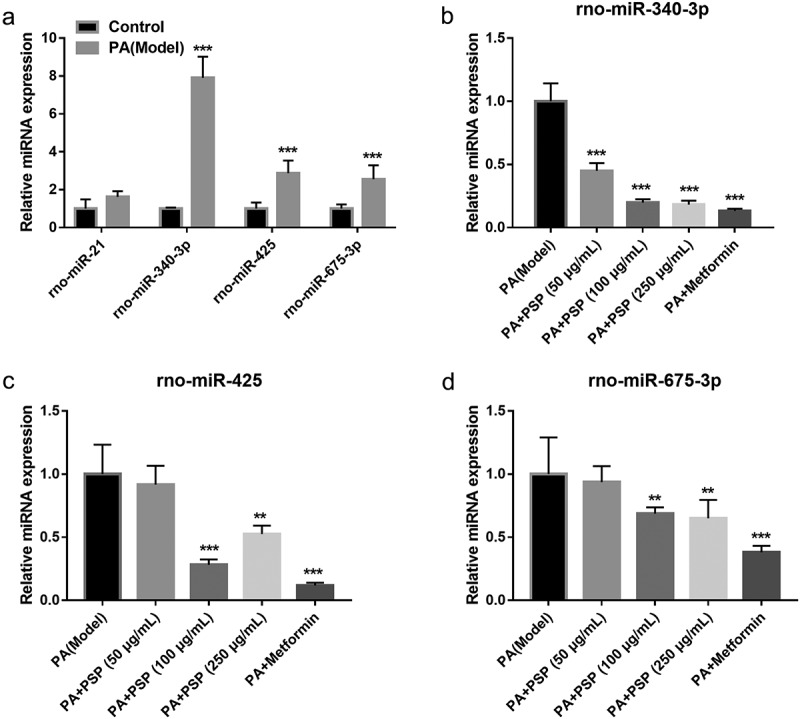
The effects of PSP on miRNAs expression in the PA-treated L6 myotubes. (a) miRNAs expression in control and PA-treated L6 myotubes as measured by qRT-PCR. ***P < 0.001. (b-d) rno-miR-340-3p (b), rno-miR-425 (c), and rno-miR-675 (d) expression as measured by qRT-PCR after PSP treatment of PA-treated L6 myotubes. **P < 0.01 and ***P < 0.001, treatment group *vs* PA group. PSP: *Polygonatum sibiricum* polysaccharide; PA: palmitic acid

Next, to explore whether PSP improves glucose metabolism via miRNAs in PA-induced L6 myotubes, the GEO database GSE68225 was selected. GSE68225 was analyzed using GEO2R, and the results showed that only four miRNAs, rno-miR-21, rno-miR-340-3p, rno-miR-425, and rno-miR-675* had significantly changed in the skeletal muscle of IR rats compared with that of normal rats (**S-**Figure 1). Therefore, this study investigated the role of these four miRNAs in PA-treated L6 myotubes. Compared with normal L6 myotubes, rno-miR-340-3p, rno-miR-675, and rno-miR-425 expression significantly increased, while rno-miR-21 expression did not significantly change in the PA-treated L6 myotubes (Figure 3a). Additionally, compared with the PA-induced L6 myotubes, rno-miR-340-3p expression levels were significantly reduced in the PA+50, 100, and 250 μg/mL PSP groups and PA+ metformin group (Figure 3b). The rno-miR-675 and rno-miR-425 expression levels were remarkably inhibited in the PA+ 100 and 250 μg/mL PSP groups, and the PA+ metformin group, whereas no change was observed in the PA+ 50 μg/mL PSP group compared to the PA model group (Figure 3c and d). This result indicated that miR-340-3p may potentially be regulated by PSP in PA-treated L6 myotubes. Therefore, miR-340-3p was chosen as an excellent candidate for subsequent experiments.

### Overexpression of miR-340-3p reverses the effect of PSP in PA-induced L6 myotubes

**Figure 4.**
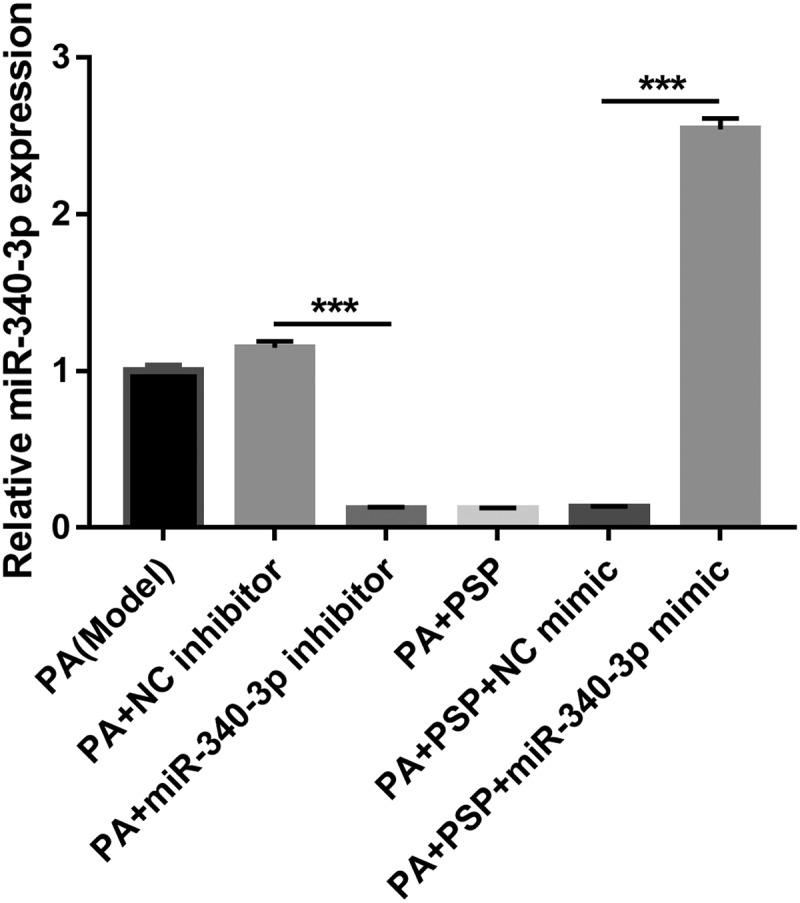
miR-340-3p expression is inhibited after transfection with miR-340-3p inhibitor, whereas it is promoted after transfection with miR-340-3p mimic

**Figure 5.**
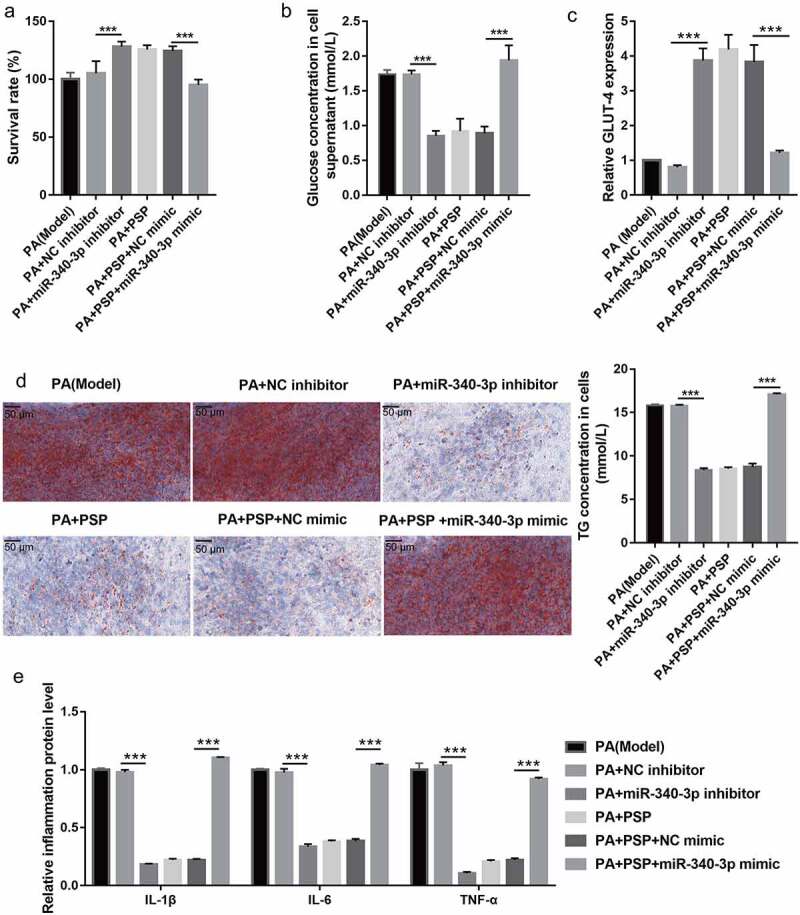
Overexpression of miR-340-3p reverses the effects of PSP on survival and glucose and TG metabolism in the insulin-resistant L6 myotubes. (a) The survival of PA-treated L6 myotubes as assessed by CCK8 assay after transfection with miR-340-3p mimic and inhibitor for 48 h. (b) The glucose concentration in the L6 myotubes supernatant as measured by glucose detect kit after stimulation with 100 nM insulin and after transfection with miR-340-3p mimic and inhibitor at 48 h. Glucose uptake efficiency was negatively correlated with glucose concentration in the supernatant. (c) The GLUT-4 expression in L6 myotubes as measured by qRT-PCR. (d) The effects of PSP on lipid accumulation as assessed by Oil Red O staining (left, ×400). The TG concentration in the L6 myotubes were measured by TG detect kit after treatment for 24 h (right). (e) The levels of IL-1β, IL-6, and TNF-α in the cell supernatant as measured by ELISA. ***P < 0.001. TG: triglyceride; PSP: *Polygonatum sibiricum* polysaccharide; PA: palmitic acid; NC: negative control; CCK8: Cell Counting Kit-8; NC: negative control

To understand whether miR-340-3p is implicated in the PSP-induced improvement of IR, miR-340-3p mimic and inhibitor were transfected into PA-induced L6 myotubes. Compared to the PA + NC inhibitor group, miR-340-3p expression level was significantly decreased in the PA + miR-340-3p inhibitor group in PA-induced L6 myotubes (Figure 4). Additionally, compared to the model group, cell survival (Figure 5a) and GLUT-4 mRNA expression (Figure 5c) were significantly increased, while the glucose concentration in the cell supernatant (Figure 5b), the TG concentration in PA-induced L6 myotubes (Figure 5d), and the levels of IL-1β, IL-6, and TNF-α in the cell supernatant (Figure 5e) were significantly inhibited in the PA + miR-340-3p inhibitor group. These results suggest that silencing miR-340-3p improves glucose uptake, TG metabolism, and inflammation in PA-induced L6 myotubes. In addition, compared to the PA+PSP+NC mimic group, miR-340-3p expression level in PA-induced L6 myotubes was significantly increased in the PA+PSP+miR-340-3p mimic group (Figure 4). Furthermore, compared to the PA+PSP+NC mimic group, cell survival (Figure 5a) and GLUT-4 mRNA expression (Figure 5c) were significantly decreased, while the glucose concentration in the cell supernatant (Figure 5b), the TG concentration in PA-induced L6 myotubes (Figure 5d), and the levels of IL-1β, IL-6, and TNF-α in the cell supernatant (Figure 5e) were significantly enhanced in the PA+PSP+miR-340-3p mimic group. These results suggest that miR-340-3p overexpression reversed the effect of PSP in PA-treated L6 myotubes.

miR-340-3p expression in PA-treated L6 myotubes after transfection with miR-340-3p mimic and inhibitor as measured by qRT-PCR. ***P < 0.001. PSP, *Polygonatum sibiricum* polysaccharide; PA, palmitic acid; NC, negative control

### Mir-340-3p targeting-inhibits IRAK3 expression

**Figure 6.**
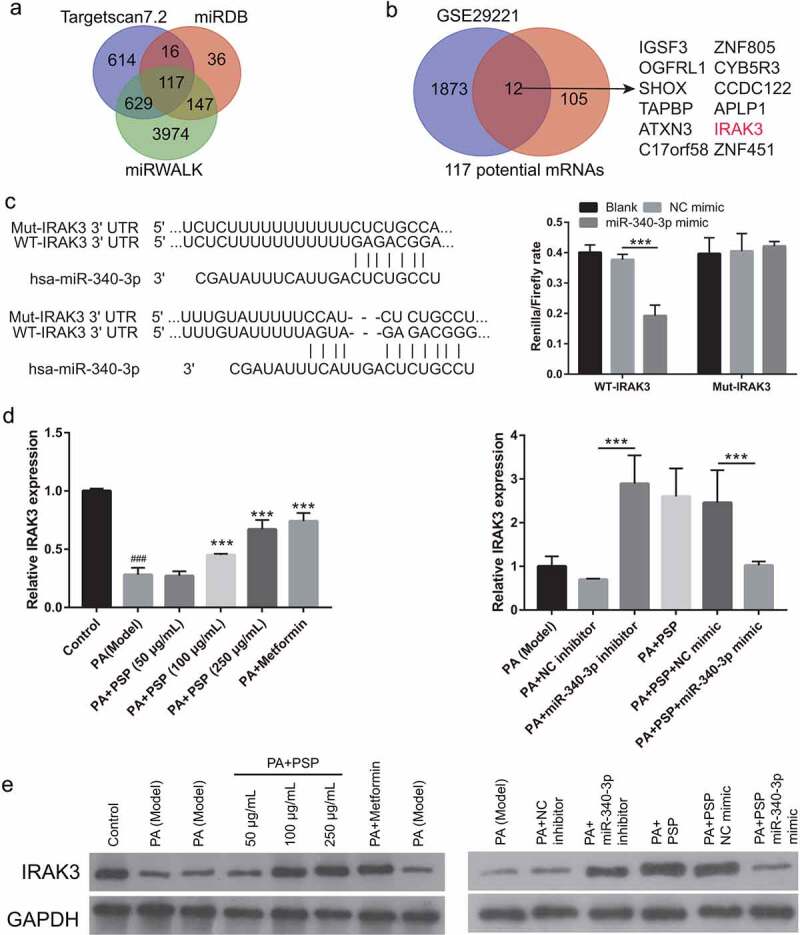
PSP treatment regulates IRAK3 expression in PA-treated L6 myotubes. (a) The target genes were analyzed by targetscan 7.0, miRDB, and miRwalk. (b) 12 mRNA intersections were found between 1885 downregulated mRNA in GSE29221 database and 117 potential target genes. (c) the binding between miR-340-3p and *IRAK3* 3′-UTR as analyzed by Luciferase reporter assay. (d) IRAK3 mRNA expression as measured by qRT-PCR. (e) IRAK3 protein level as measured by Western blot. ^###^P < 0.001 vs control group; ***P < 0.001 vs model group

Finally, to further understand the target genes regulated by miR-340-3p, the potential target genes were analyzed using Targetscan 7.0, miRDB, and miRwalk website and verified by luciferase reporter assay. The results showed that 117 mRNAs were potential target mRNAs of miR-340-3p (Figure 6a). Additionally, 1885 downregulated mRNAs were found in the GSE29221 database, which had 12 mRNA intersections with 117 potential target genes (Figure 6b). The fold changes in the expression of 12 mRNAs are shown in **Supplementary Table 1**. A previous study found that IRAK3 expression in liver tissue was related to diabetes development [24]. Therefore, we selected IRAK3 for further studies. Luciferase reporter assay results showed that miR-340-3p can bind to the IRAK3 3′-UTR (Figure 6c). Compared with that of the control group, IRAK3 mRNA expression and protein levels in the model group were reduced (Figures 6d and e). Furthermore, compared with that of the model group, IRAK3 mRNA expression and protein levels were enhanced in the 100 and 250 μg/mL PSP and metformin treatment groups (Figures 6d and e). Additionally, compared with that of the PA+NC inhibitor group, IRAK3 mRNA expression and protein levels in the PA+miR-340-3p inhibitor group were increased (Figures 6d and e). Compared to the PA+PSP+NC mimic group, IRAK3 mRNA expression and protein levels in the PA+PSP+ miR-340-3p mimic group were decreased (Figure 6d and e). These results suggest that miR-340-3p targeting inhibits IRAK3 expression and that PA treatment inhibits IRAK3 expression, whereas treatment with 100 and 250 μg/mL PSP enhances IRAK3 expression.

## Discussion

In recent years, the incidence of T2DM has continued to rise due to dietary and lifestyle habits, especially in Asian countries, including China and India [25]. IR acts as a precursor of T2D development and still lacks efficient therapy. Excess lipid accumulation in muscle, which is induced by high concentrations of circulating saturated fatty acids, including PA, leads to IR [26,27]. In this study, L6 myotubes were treated with PA stimulation to establish an IR cell model. PA stimulation reduced cell survival, suppressed glucose and lipid metabolism, and increased inflammation in L6 myotubes (Figure 2).

PSP is a natural polysaccharide with multiple pharmacological activities, for example, antioxidant, anti-aging, and anti-inflammatory effects, as well as blood glucose and blood lipid regulation activities [3–5]. It exhibits the ability to decrease fasting blood glucose, total cholesterol, and triglyceride levels, and islet cell apoptosis in diabetic rats [9,28]. Moreover, PSP could promote glucose uptake in high‑glucose‑ and high‑insulin‑induced 3T3‑L1 adipocytes [10]. These results suggest that PSP is a potential drug for improving IR and T2DM. Similar to these results, we demonstrated that PSP improves IR by promoting cell survival, improving glucose and lipid metabolism, and inhibiting inflammation in PA-treated L6 myotubes (Figure 2).

Recent studies have also found that miRNAs play an important role in the development and progression of diabetes. miRNAs are involved in cell survival, blood glucose and lipid metabolism, IR, and islet β cell damage and dysfunction [29,30]. miR-340-3p is a multifunctional miRNA that is involved in cell survival, apoptosis, and differentiation [31,32], suggesting that miR-340-3p participates in the pathological processes of human diseases. However, the effect of miR-340-3p on IR in skeletal muscle remains unknown. In this study, we found that miR-340-3p expression was significantly increased in L6 myotubes after PA stimulation, and miR-340-3p knockdown promoted cell survival, improved glucose and lipid metabolism, and inhibited inflammation in the PA-treated L6 myotubes, which played an important role in improving IR in skeletal muscles (Figure 3a and Figure 5). Subsequently, miR-340-3p expression was significantly inhibited in PA-treated L6 myotubes after PSP treatment, and miR-340-3p overexpression reversed the improvement effects of PSP (Figure 3b and Figure 5), suggesting that PSP plays an important role in PA-treated L6 myotubes by inhibiting miR-340-3p.

Using GEO2R to analyze the GSE29221 dataset in this study, we found that IRAK3 mRNA expression was downregulated in diabetic patients. In previous studies, the GSE29221 dataset was used to analyze the key genes involved in diabetes and diabetes complications [21,33,34]. Our purpose was to analyze the genes involved in PSP and miR-340-3p regulated glucose uptake by skeletal muscle cells in diabetes, thus being different from the purpose of previous studies. IRAK3 is an important negative regulator of TLR-mediated cell signaling, which can prevent the formation of IRAK–TNF receptor-associated factor-6 complexes and silence the NF-κB signaling pathway [35]. IRAK3, a key inhibitor of inflammation, promotes diabetes development [24,36,37]. Moreover, IRAK3 mRNA expression and protein levels were inhibited by PA treatment, and this effect was reversed by PSP treatment. miR-340-3p targeting inhibited IRAK3 mRNA expression and protein levels (Figure 6). These results suggest that PSP improves the IR in skeletal muscle cells by regulating the miR-340-3p/IRAK3 axis.

## Conclusions

**Figure 7.**
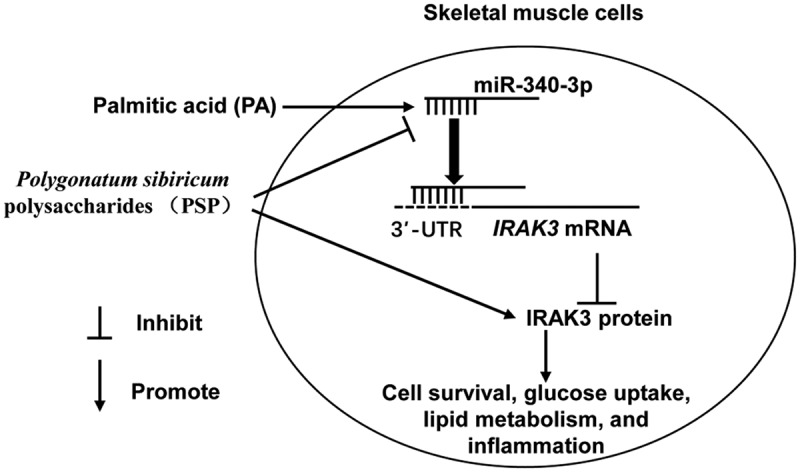
Illustration of mechanistic action of PSP in L6 skeletal muscle cells. PA stimulation significantly suppressed cell survival and glucose uptake while increased lipid accumulation and inflammation in L6 myotubes. PSP improve the PA effect in skeletal muscle cells by inhibiting miR-340-3p/IRAK3 axis

PSP improved the PA-induced inhibition of cell survival, inflammation, and glucose uptake in skeletal muscle cells by inhibiting the miR-340-3p/IRAK3 axis. Additionally, PSP treatment inhibited miR-340-3p expression and enhanced IRAK3 expression in PA-induced L6 myotubes. *IRAK3* is a target gene of miR-340-3p. Taken together, PSP could be considered a potential therapeutic agent for the treatment of IR (Figure 7).

## Supplementary Material

Supplemental Material

## Data Availability

All data during the study appear in the submitted article.
