# Supplementary material for: Polygonatum sibiricum polysaccharides (PSP) improve the palmitic acid (PA)-induced inhibition of survival, inflammation, and glucose uptake in skeletal muscle cells
Source: Bioengineered. 2021 Dec 7;12(2):10147–59. doi: 10.1080/21655979.2021.2001184 (PMC8810107; doi:10.1080/21655979.2021.2001184)
Supplement: Supplemental Material [file KBIE_A_2001184_SM6480.zip › supplementary/Supplementary materials.docx]

**Supplementary materials:**

**Supplementary Figure 1** changes in miRNA expression in skeletal muscle of normal rats (n=6) and insulin-resistant rats (n=6) was analyzed by GEO2R analysis (GSE68225 database).

**Supplementary Figure 2:** Full-length blots are presented. IRAK3 protein level as measured by western blot after treatment.

**Supplementary Figure 3:** Full-length blots are presented. IRAK3 protein level as measured by western blot after transfection.
