# Supplementary material for: Polygonatum sibiricum polysaccharides (PSP) improve the palmitic acid (PA)-induced inhibition of survival, inflammation, and glucose uptake in skeletal muscle cells
Source: Bioengineered. 2021 Dec 7;12(2):10147–59. doi: 10.1080/21655979.2021.2001184 (PMC8810107; doi:10.1080/21655979.2021.2001184)
Supplement: Supplemental Material [file KBIE_A_2001184_SM6480.zip › supplementary/Supplementary Table 1.docx]

**Supplementary Table 1** Significantly changed-mRNAs between biopsy samples of skeletal muscle of three male patients of type 2 diabetes and three non-diabetic male patients was analyzed by GEO2R analysis (GSE29221 database)

| ID | logFC | P. Value |
| --- | --- | --- |
| STX2 | -1.118 | 0.006 |
| MBOAT1 | -1.552 | 0.001 |
| ZNF805 | -1.567 | 0.047 |
| SLC44A1 | -1.004 | 0.002 |
| CPM | -1.829 | 0.004 |
| IRAK3 | -1.780 | 0.021 |
| GYG2 | -2.224 | 0.013 |
| BNIPL | -1.412 | 0.019 |
| PLCXD1 | -1.742 | 0.003 |
| OGFRL1 | -0.740 | 0.006 |
| RABL2A | -1.575 | 0.032 |
| VHL | -1.059 | 0.001 |
