# Supplementary figures and images for: Polygonatum sibiricum polysaccharides (PSP) improve the palmitic acid (PA)-induced inhibition of survival, inflammation, and glucose uptake in skeletal muscle cells
Source: Bioengineered. 2021 Dec 7;12(2):10147–59. doi: 10.1080/21655979.2021.2001184 (PMC8810107; doi:10.1080/21655979.2021.2001184)

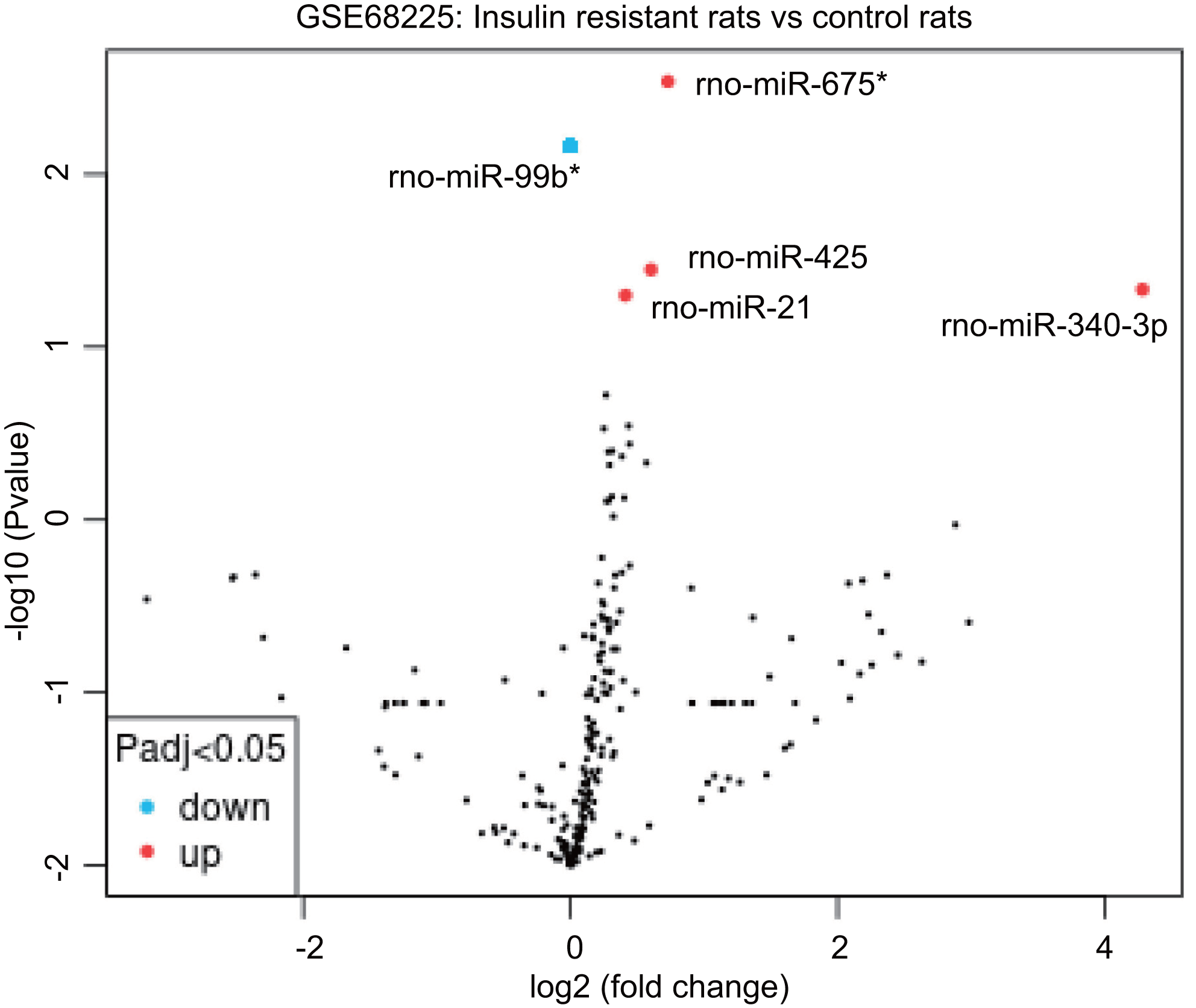

Supplement: Supplemental Material [file KBIE_A_2001184_SM6480.zip › supplementary/Supplementary Figure 1 (3).tif]
